# Advancing the field of health systems research synthesis

**DOI:** 10.1186/s13643-015-0080-9

**Published:** 2015-07-10

**Authors:** Etienne V. Langlois, Michael K. Ranson, Till Bärnighausen, Xavier Bosch-Capblanch, Karen Daniels, Fadi El-Jardali, Abdul Ghaffar, Jeremy Grimshaw, Andy Haines, John N. Lavis, Simon Lewin, Qingyue Meng, Sandy Oliver, Tomás Pantoja, Sharon Straus, Ian Shemilt, David Tovey, Peter Tugwell, Hugh Waddington, Mark Wilson, Beibei Yuan, John-Arne Røttingen

**Affiliations:** Alliance for Health Policy and Systems Research, World Health Organization, 20 avenue Appia, 1211 Geneva, Switzerland; World Bank Group, 3 Chemin Louis-Dunant, Post Office Box 66 CH, 1211 Geneva, Switzerland; Department of Global Health and Population, Harvard School of Public Health, 665 Huntington Avenue, Boston, MA 02115 USA; Wellcome Trust Africa Centre for Health and Population Studies, University of KwaZulu-Natal, R618 en route to Hlabisa, Somkhele A2074 Rd, Mtubatuba, 3935 South Africa; Swiss Tropical and Public Health Institute, Socinstrasse 57, 4051 Basel, Switzerland; University of Basel, Petersplatz 1, 4003 Basel, Switzerland; Health Systems Research Unit, South African Medical Research Council, Francie van Zijl Drive Parowvallei, Cape , PO Box 19070 , 7505, Tygerberg South Africa; Department of Health Management and Policy, Center for Systematic Reviews of Health Policy and Systems Research (SPARK), Faculty of Health Sciences, Van Dyck Hall, American University of Beirut, PO Box 11-0236, Riad El-Solh, Beirut, 1107 2020 Lebanon; Ottawa Hospital Research Institute & Department of Medicine, University of Ottawa, 725 Parkdale Ave, Ottawa, ON K1Y 4E9 Canada; Departments of Social and Environmental Health Research and of Population Health, London School of Hygiene & Tropical Medicine, Keppel Street, London, WC1E 7HT UK; Department of Clinical Epidemiology & Biostatistics, Department of Political Science, and McMaster Health Forum, PPD/CHEPA, McMaster University, 1280 Main Street West, CRL-209, Hamilton, ON L8S 4K1 Canada; Cochrane Effective Practice and Organisation of Care Group (EPOC) Satellite, Norwegian Knowledge Centre for the Health Services, Box 7004, St. Olavs plass, 0130 Oslo, Norway; China Centre for Health Development Studies, Peking University, PO 505, XueYuan Road 38, Haidian District, Beijing, 100191 China; Evidence for Policy and Practice Information and Co-ordinating Centre (EPPI-Centre), UCL Institute of Education, University College London, 20 Bedford Way, London, WC1H 0AL UK; Departamento de Medicina Familiar, Escuela de Medicina, Pontificia Universidad Católica de Chile, Avenida Libertador Bernardo O Higgins 340, Santiago, Chile; St. Michael’s hospital, Li Ka Shing Knowledge Institute, 30 Bond St, Toronto, ON M5B 1W8 Canada; Behaviour and Health Research Unit, School of Clinical Medicine, University of Cambridge, Box 113 Cambridge Biomedical Campus Forvie Site, Robinson Way, Cambridge, CB2 0SR UK; Cochrane, St Albans House, 57-59 Haymarket, London, SW1Y 4QX UK; Department of Medicine, Centre for Global Health, WHO Collaborating Centre for Knowledge Translation and Health Technology Assessment in Health Equity, Institute of Population Health, Bruyère Research Institute, University of Ottawa, 85 Primrose Avenue, Office 302, Ottawa, ON K1R 6M1 Canada; International Initiative for Impact Evaluation, 36 Gordon Square, London, WC1H 0PD UK; Department of Health Management and Health Economics, Faculty of Medicine, Institute of Health and Society, University of Oslo, Postboks 1089, Blindern, 0317 Oslo, Norway; Division of Infectious Disease Control, Norwegian Institute of Public Health, PO Box 4404, , N-0403 Oslo, Norway

**Keywords:** Evidence synthesis, Health systems research, Health policy, Systematic reviews, Decision-making

## Abstract

Those planning, managing and working in health systems worldwide routinely need to make decisions regarding strategies to improve health care and promote equity. Systematic reviews of different kinds can be of great help to these decision-makers, providing actionable evidence at every step in the decision-making process. Although there is growing recognition of the importance of systematic reviews to inform both policy decisions and produce guidance for health systems, a number of important methodological and evidence uptake challenges remain and better coordination of existing initiatives is needed. The Alliance for Health Policy and Systems Research, housed within the World Health Organization, convened an Advisory Group on Health Systems Research (HSR) Synthesis to bring together different stakeholders interested in HSR synthesis and its use in decision-making processes. We describe the rationale of the Advisory Group and the six areas of its work and reflects on its role in advancing the field of HSR synthesis. We argue in favour of greater cross-institutional collaborations, as well as capacity strengthening in low- and middle-income countries, to advance the science and practice of health systems research synthesis. We advocate for the integration of quasi-experimental study designs in reviews of effectiveness of health systems intervention and reforms. The Advisory Group also recommends adopting priority-setting approaches for HSR synthesis and increasing the use of findings from systematic reviews in health policy and decision-making.

## Background

Health policymakers and managers (henceforth decision-makers), as well as other stakeholders (professional leaders, civil society representatives) involved in health systems worldwide, routinely face difficult decisions around improving health care and promoting equity. Ideally, decisions and recommendations should be informed by the best available research evidence, which typically comes from systematic reviews of research [1–3]. Systematic reviews of health systems evidence can be of great help to decision-makers, providing actionable evidence at every step in the decision-making process [4]. These steps require different types of systematic reviews to address: (i) clarifying problems and their causes; (ii) assessing potential policy and programmatic options, including their effectiveness; and (iii) identifying implementation considerations (Table 1) [5]. Systematic reviews are also valuable to identify priority questions for new primary Health Systems Research (HSR).

**Table 1:** How systematic reviews can inform different steps in policy-making processes (adapted from Lavis 2009) [5]

Steps in policy-making	Policy question	Types of systematic reviews
Clarifying the problem and its causes	Need for intervention: what is the nature, magnitude and appropriate framing of the problem?	Reviews of observational and qualitative studies
Assessing potential policy and programmatic options	What is the appropriate set of options to address the problem and what are the effects, cost-effectiveness and acceptability of these options?	Reviews of effectiveness studies, economic evaluations, studies of views and experiences, process evaluations
Identifying	What are the potential barriers to the successful implementation of the options and potential windows of opportunity?	All of the above
Implementation		
Considerations		

The Alliance for Health Policy and Systems Research (“Alliance”), a partnership housed within the World Health Organization (WHO), is pioneering support for strengthening capacity for synthesis of health policy and systems research through the establishment of systematic review centres in low- and middle-income countries (LMICs). The need for such a resource is especially acute in these settings due to the limited capacity of individuals, teams, organisations and knowledge systems to support the production and use of systematic reviews [6]. To date, the Alliance has supported centres in Bangladesh, Chile, China, Lebanon, South Africa and Uganda. The focus of the centres is on reviews addressing questions of how to improve the performance of health systems, and more than 20 systematic reviews and review protocols have been produced so far (for examples, see [7–13]).

The work of the Alliance centres complements that of other recent global initiatives. The Evidence-Informed Policy Network (EVIPNet) has actively promoted partnerships at the country level between policymakers, researchers and civil society in order to facilitate both policy development and policy implementation informed by the best scientific evidence available [14]. Similarly, the Task Force on Developing Health Systems Guidance, convened by WHO, considered how evidence should be translated into guidance to inform policies on health systems and improve the delivery of public health and health systems interventions [1, 2, 15].

Although there is growing recognition of the importance of systematic reviews to both inform policy decisions and produce guidance for health systems, a number of important methodological challenges remain and there is a need to better understand the processes supporting the transparent use of review findings in complex health system decisions. In addition, there is a multiplicity of initiatives in the field and better coordination is needed. The Alliance therefore convened an Advisory Group on Health Systems Research Synthesis (“Advisory Group”) to bring together different collaborations, groups and institutions interested in HSR synthesis and its use in decision-making processes [16].

In this paper, we outline the rationale of the Advisory Group, the six areas of work steering its activities and the guidance it provides to the Alliance and beyond; the paper also reflects on challenges in producing complex HSR syntheses and fostering their use in health systems decision-making.

### Rationale for the advisory group

Since 2009, a series of consultative meetings led to the development of recommendations to improve international collaboration on HSR synthesis, with a focus on LMIC needs and capacity building (Table 2). The consultations focused on the following: (i) the benefits and challenges associated with collaborative efforts to synthesise and translate health systems evidence and (ii) the range of approaches to evidence syntheses needed to address the types of questions raised in decision-making processes for strengthening health systems (Table 1).

**Table 2:** Evolution of the Advisory Group on Health Systems Research Synthesis

Date	Location	Activity
November 2009	Havana (Cuba)	Organized Session during the Global Forum for Health Research and initiation of a consultative process on HSR synthesis collaboration
October 2010	London (UK)	Ad Hoc Working Group on HSR Synthesis meeting at the UK Department for International Development (DFID) and recommendation to establish an Advisory Group on HSR Synthesis to facilitate further discussions and coordinate efforts
November 2010	Montreux (Switzerland)	Session at the First Global Symposium on Health Systems Research: development of a set of recommendations to inform the activities of the Advisory Group on HSR Synthesis
Currently	Beijing, ChinaCape Town, South Africa	Quarterly meetings of the Advisory Group, including meetings alongside the Global Symposia on Health Systems Research in Beijing, China (2012) and Cape Town, South Africa (2014).

The consultative meetings also revealed a landscape of research synthesis in which the focus and activities of organisations varied in terms of the following: (1) content (health systems or welfare practices and policies), (2) research questions (e.g., effectiveness, views and experiences), (3) geographical focus (high-income countries (HIC) or LMIC)), and (4) capacity building (for supply and/or demand for reviews). The Advisory Group was established to play a central role in advancing the science of HSR synthesis and knowledge translation by developing and strengthening networks among individuals, groups, institutions and collaborations that have an interest in this field. Participating organisations include Cochrane and The Campbell Collaboration, numerous academic institutions and governmental and nongovernmental organisations (e.g., the Norwegian Institute of Public Health and the International Initiative for Impact Evaluation (3ie)).

## Discussion

### Areas of work of the advisory group

#### Area 1 Advancing the science of HSR synthesis by developing and strengthening networks between individuals and institutions and collaborations and groups that have an interest in HSR synthesis and translation

The Advisory Group has evolved to include participants from 17 organizations/networks who convene once every 3 to 4 months to discuss current challenges and advances related to both producing systematic reviews and drawing on them for guidance on strengthening health systems. The group shares information, identifies where further methodological work or training is needed and builds cross-institutional links, and thereby, with its anchoring at the Alliance and WHO, serves as a global focal point for coordination around the issues.

#### Area 2 Providing support, information sharing and coordination related to setting priorities for HSR synthesis regionally and globally and increasing capacity building in HSR synthesis and translation (particularly in relation to LMICs)

The Advisory Group, together with the Alliance more broadly, has contributed to setting priorities for health systems research on a global scale [17, 18]. The systematic review centres funded by the Alliance have developed priority-setting approaches for HSR syntheses and, in doing so, have implemented different mechanisms to engage with decision-makers, to increase the likelihood of the synthesised evidence being applied in practice. For instance, the South African Medical Research Council developed an electronic survey addressed to both academics and practitioners/policymakers, in order to identify key HSR issues to be reviewed. Results of the survey informed a preselection phase in which existing systematic reviews relevant to the HSR issues identified were summarised. This process informed the ranking of a set of the highest priority questions by a steering committee and advisory group, which included policymakers at national and regional levels. Another example is the Center for Systematic Reviews of Health Policy and Systems Research (SPARK) at the American University of Beirut in Lebanon, which is developing a tool for prioritizing questions for HSR systematic reviews. Although priority-setting approaches exist for some types of research [19, 20], little work addresses how to engage policymakers and other stakeholders in prioritizing research [21] and there is no published framework to guide priority setting for health policy and systems research synthesis.

Limited capacity and issues of financial sustainability of groups in LMICs that undertake systematic reviews are further important challenges identified by the Advisory Group. For example, how can these groups secure core funding, ensure the availability of specialised resources such as information scientist support and statistical tools, strengthen their capacity to undertake the wide range of reviews needed to inform health system decisions and improve their engagement with decision-makers in the review process? Discussions on these issues contributed to the development of the new Global Evidence Synthesis Initiative (GESI), led by an international collaborating group of organisations^1^ and convened by Cochrane. It is anticipated that GESI will coordinate and support the development of capacities for conducting systematic reviews (not necessarily limited to health systems evidence or even the health sector) in LMICs, building on the resources and networks of the collaborating organisations.

Another important challenge identified by the Advisory Group relates to strengthening the capacity of decision-makers and stakeholders to access, assess and apply HSR evidence in decision-making and to create a “user-pull” for HSR evidence. The Advisory Group advocates for and supports the development of user-friendly summaries of HSR syntheses and recognises the importance of sensitization workshops for users of HSR syntheses, institutionalised mechanisms for researcher-user interactions and knowledge translation activities. The last involves using knowledge translation tools such as evidence briefs for policy and briefing notes and convening dialogue sessions with decision-makers and stakeholders.

#### Area 3 Expanding the range of study designs that can be included in reviews of the effectiveness of HSR interventions

For a number of areas within the field of HSR, evidence from quasi-experimental studies forms a substantive component of the overall evidence base for questions on the impacts of interventions. Evidence from quasi-experimental studies may be particularly important for reviews of interventions or exposures whose effects are not, often for practical reasons, easily amenable to measurement using designs based on random assignment of participants or facilities to comparison groups under the direct control of researchers. Quasi-experimental studies, such as regression discontinuity designs, [22, 23] interrupted time series [24] and instrumental variable analyses [25], offer potential for inferences to be made about the causal effects of health systems interventions and reforms. These inferences can be as valid as those derived from randomised controlled trials—without intervening externally in the health system. Quasi-experimental studies thus allow the study of intervention and reform effectiveness in their natural setting.

However, efforts to integrate quasi-experimental studies into effectiveness review frameworks in both HICs and LMICs are, in general, at an early stage of development [26–28]. This is in part because methods and standards for incorporating evidence from quasi-experimental studies are underdeveloped for most fields of study, including HSR [29]. Review authors therefore do not always include these study designs when it might be appropriate to do so. For instance, two recent studies of systematic reviews of health systems evidence found wide variation in their inclusion of quasi-experimental studies and suggested that more work is needed on when it may be useful to do so [29, 30].

With the Harvard School of Public Health, the Alliance organized a workshop in 2013 focused on the following: (1) establishing a taxonomy of quasi-experimental study designs, (2) developing guidelines for systematic review authors on incorporating quasi-experimental studies in reviews, (3) devising strategies to build institutional capacity to support the wider inclusion of quasi-experimental studies in reviews, (4) establishing approaches to translate evidence from quasi-experimental studies for policymakers, and (5) strengthening partnerships between researchers in HICs and researchers in LMICs.

One of the outputs of the workshop and the background work commissioned by the Alliance is the identification of five quasi-experimental study designs frequently employed in health systems research: natural experiments, instrumental variable analyses, regression discontinuity analyses, interrupted times series studies and difference studies including controlled before-and-after designs, difference-in-difference designs and fixed effects analyses of panel data [31].

#### Area 4 Piloting a system for producing policy relevant syntheses/systematic reviews of HSR addressing questions other than effectiveness

Systematic reviews addressing the full range of health system issues may be useful for decision-making (Table 1). However, guidance around syntheses for questions other than effectiveness of interventions remains limited. Further guidance is needed on templates for reporting results, tools to assess confidence in review results and institutional mechanisms to facilitate evidence uptake.

The EPPI Centre is leading a study, funded by the Alliance, on supporting policy-relevant systematic reviews for health systems. Interviews with policymakers and systematic reviewers have emphasised the importance of connecting these two groups and supporting their mutual engagement with formalised procedures and structures, so as to ensure that their motivations for producing and using systematic reviews are aligned in terms of the urgency and generalizability of products [32].

In addition, members of the Advisory Group are conducting scoping reviews to assess the most appropriate knowledge synthesis methods to answer different types of research questions [33, 34]. These reviews aim to identify, define and classify emerging knowledge synthesis methods.

#### Area 5 Advocating for and supporting a common global database for all types of systematic reviews of HSR

The Advisory Group acknowledges the utility and promotes the use of repositories of evidence synthesis in this field, such as Health Systems Evidence (HSE) (www.healthsystemsevidence.org), a database for all types of systematic reviews of HSR. In this database, systematic reviews, and links to their included primary studies, are complemented by a range of other policy-relevant documents addressing health systems, including evidence briefs for policymakers, overviews of systematic reviews, systematic review protocols, economic evaluations and descriptions of health system reforms. The database is available in Arabic, Chinese, English, French, Portuguese, Russian and Spanish; it can be searched using country, region and LMIC filters; it includes links to user-friendly summaries written by other groups. These features make the database a particularly powerful resource for health system decision-makers, stakeholders and researchers. HSE also now includes the beta version for an Intergovernmental Organizations’ Health Systems Documents Portal, developed as a complete inventory of all policy-relevant WHO documents about health systems. Another useful repository is PDQ-Evidence (http://www.pdq-evidence.org), which links systematic reviews of HSR and other health research, overviews of reviews and their included primary studies.

These databases are useful in identifying existing reviews that could inform decisions on specific HSR issues and should also be consulted at the planning stages for new primary health system research and for reviews. This would allow identification of gaps in the HSR literature and key areas for further HSR, including implementation research (Table 1).

#### Area 6 Advocating for and supporting prospective registration of all protocols for systematic reviews when they are planned

Prospective registration of systematic reviews is an important step in reducing duplication of effort, fostering collaboration on conducting reviews and enabling comparison of the completed review with what was planned originally. International prospective register of systematic reviews (PROSPERO), the international prospective register of systematic reviews (http://www.crd.york.ac.uk/prospero), was established for this purpose but initially included only reviews of effectiveness. The Advisory Group therefore initiated discussions with PROSPERO regarding how the full range of health systems systematic reviews (Table 1) could be accommodated. This feedback, as well as findings from a survey of users [35], led to modifications to provide a system sufficiently flexible to accommodate a range of review types.

## Conclusion

The Advisory Group continues to identify and address key challenges faced by those undertaking HSR synthesis, including issues relevant to strengthening dialogue and collaboration between producers and users of evidence syntheses, as well as the uptake of findings from these reviews. A number of challenges remain in producing and using HSR evidence synthesis in relation to (i) the research domain, for instance human and financial resource constraints for conducting reviews and limited open access to and functionality of databases; (ii) the policy domain, including limited access to HSR systematic reviews and difficulties in assessing both how well evidence syntheses were conducted and how much confidence to have in their findings; and (iii) optimising the linkages between these domains to foster the uptake of findings into policy-making, for instance the alignment of review questions with policy needs and timeliness in the production and uptake of HSR synthesis.

Further work of the Advisory Group aims to address these challenges, including capacity development and methods development across these domains. Ongoing work by members of the Advisory Group includes developing norms and standards for qualitative and mixed-methods reviews, building on existing resources [36, 37] and designing approaches to assess how much confidence to place in evidence from different types of health systems research synthesis. For example, a team of methodologists is developing a tool to assess how much confidence to place in evidence from reviews of qualitative research (the CERQual tool) [38, 39].

We argue that early engagement with decision-makers is critical for prioritizing, planning and conducting HSR systematic reviews and for enhancing the relevance and uptake of review findings. In engaging with decision-makers, special attention should be given to the challenge of translating priority policy issues into reviewable HSR questions. Funders of initiatives to strengthen health systems are also a key target audience of the Advisory Group. When designing and funding such initiatives in LMICs, we would suggest that funders take into consideration both available policy-relevant HSR syntheses and the need to build capacity for HSR synthesis.

Those conducting reviews should give more consideration, when it is appropriate to do so, to evidence from quasi-experimental studies on the impacts of health systems interventions. Following from this, further development of the methods and standards for incorporating quasi-experimental evidence into reviews is needed. Complementary to developing standards for reviewing is the work of the EQUATOR (Enhancing the QUAlity and Transparency Of health Research) initiative, which seeks to improve the reliability and value of health research by promoting transparent and accurate reporting of studies and reviews [40]. Given the complexity of health systems’ decision-making processes, we also need to undertake systematic reviews addressing the full range of HSR issues, including questions other than the effectiveness of health system interventions.

Overall, the focus on collaborative priority setting for research, judicious choice of study designs and relying on systematic reviews rather than single studies for making decisions about health systems set this work alongside the movement advocating less waste and greater value in biomedical research [41, 42]. We argue that greater cross-institutional collaboration is needed to advance the science and practice of health systems research synthesis. Users of HSR syntheses also need to be assisted in developing their awareness and ability to identify, assess and use review findings in health systems decision-making.

These efforts need continued collaboration and coordination across different organizations, networks and actors. We believe a coordination mechanism like the Advisory Group can fulfil the need for aligning interests, methods and approaches and for securing interest and investments in the field of health systems research synthesis globally.

More information on the activities of the Advisory Group is available at http://www.who.int/alliance-hpsr/projects/hsrsynthesis/en/index.html

## Abbreviations

3ie: International Initiative for Impact Evaluation
EPOC: Cochrane Effective Practice and Organisation of Care Group
EPPI-Centre: Evidence for Policy and Practice Information and Co-ordinating Centre
EVIPNet: Evidence-Informed Policy Network
GESI: Global Evidence Synthesis Initiative
HIC: high-income countries
HPSR: Health Policy and Systems Research
HSE: Health Systems Evidence
HSR: Health Systems Research
LMIC: low- and middle-income countries
PROSPERO: international prospective register of systematic reviews
WHO: World Health Organization

## Footnotes

1 Alliance for Health Policy and Systems Research, The Campbell Collaboration, Cochrane, Evidence for Policy and Practice Information and Co-ordinating Centre (EPPI-Centre) and International Initiative for Impact Evaluation (3ie).
